# Decompression in Chiari Malformation: Clinical, Ocular Motor, Cerebellar, and Vestibular Outcome

**DOI:** 10.3389/fneur.2017.00292

**Published:** 2017-06-22

**Authors:** Nicolina Goldschagg, Katharina Feil, Franziska Ihl, Siegbert Krafczyk, Mathias Kunz, Jörg Christian Tonn, Michael Strupp, Aurelia Peraud

**Affiliations:** ^1^German Center for Vertigo and Balance Disorders, University Hospital Munich, Munich, Germany; ^2^Department of Neurology, University Hospital Munich, Munich, Germany; ^3^Department of Neurosurgery, University Hospital Munich, Munich, Germany

**Keywords:** Chiari malformation, suboccipital decompression, cerebellar tonsil, ocular motor function, cerebellum

## Abstract

**Background:**

Treatment of Chiari malformation can include suboccipital decompression with resection of one cerebellar tonsil. Its effects on ocular motor and cerebellar function have not yet been systematically examined.

**Objective:**

To investigate whether decompression, including resection of one cerebellar tonsil, leads to ocular motor, vestibular, or cerebellar deficits.

**Patients and methods:**

Ten patients with Chiari malformation type 1 were systematically examined before and after (1 week and 3 months) suboccipital decompression with unilateral tonsillectomy. The work-up included a neurological and neuro-ophthalmological examination, vestibular function, posturography, and subjective scales. Cerebellar function was evaluated by ataxia rating scales.

**Results:**

Decompression led to a major subjective improvement 3 months after surgery, especially regarding headache (5/5 patients), hyp-/dysesthesia (5/5 patients), ataxia of the upper limbs (4/5 patients), and paresis of the triceps and interosseal muscles (2/2 patients). Ocular motor disturbances before decompression were detected in 50% of the patients. These symptoms improved after surgery, but five patients had new persisting mild ocular motor deficits 3 months after decompression with unilateral tonsillectomy (i.e., smooth pursuit deficits, horizontally gaze-evoked nystagmus, rebound, and downbeat nystagmus) without any subjective complaints. Impaired vestibular (horizontal canal, saccular, and utricular) function improved in five of seven patients with impaired function before surgery. Posturographic measurements after surgery did not change significantly.

**Conclusion:**

Decompression, including resection of one cerebellar tonsil, leads to an effective relief of patients’ preoperative complaints. It is a safe procedure when performed with the help of intraoperative electrophysiological monitoring, although mild ocular motor dysfunctions were seen in half of the patients, which were fortunately asymptomatic.

## Introduction

Chiari malformation comprises a group of pathological entities characterized by the presence of anatomical deformities of the cerebellum and brainstem. Typical symptoms include occipital headaches, which are exacerbated by coughing; motor and/or sensory disturbances especially of the upper limb; and clumsiness (1, 2).

Current treatment includes a decompression of the suboccipital region to enlarge the diameter of the foramen magnum and resection of the posterior arch of C1. But there has been much critical discussion of whether one should open the arachnoid and remove the cerebellar tonsils as well (3). The postoperative risks of this procedure seem to be higher, including pseudomeningocele and hygroma formation, meningitis, and headaches (2). On the other hand, sufficient decompression of the brainstem is more likely when the arachnoid and the tonsils are manipulated (1, 4) (details in Data Sheet S1 in Supplementary Material). Data about clinical outcome after surgical treatment vary due to different surgical methods, patient cohorts, and symptoms. Clinical improvement and improvement in daily life have been described in different studies in 50–75% of patients (2, 3). Ocular motor dysfunctions improved in 75–100% of patients (5–7).

Experiences with cerebellar lesions, i.e., due to ischemic infarcts, teach us that tonsil lesions can lead to significant ocular motor deficits causing disabling vertigo (8). However, the effect of unilateral tonsillectomy on ocular motor and cerebellar function has not yet been examined systematically. The major aim of our study was to evaluate whether the surgical procedure of suboccipital decompression, including reduction of one cerebellar tonsil, leads to ocular motor, vestibular, or cerebellar deficits in patients with Chiari malformation type I.

## Materials and Methods

### Clinical Examination of Patients

Fourteen consecutive patients with radiological evidence of Chiari malformation type 1 were screened for this study in the Departments of Neurology and Neurosurgery of the University Hospital Munich. Three patients had mild clinical signs and were treated conservatively. Eleven underwent surgical intervention with resection or reduction of one cerebellar tonsil and were included in the study. One patient was lost to follow-up.

Patients were examined before and at two time points after surgical intervention: one directly after and one in follow-up approximately 3 months after surgery. Patients received a standardized questionnaire for typical complaints. Patients were asked about headache or neck pain (type and provoking factors), gait unsteadiness, vertigo or dizziness (type and provoking factors), visual problems, audiological problems, motor signs, sensory deficits, and lower cranial nerve deficits.

The examination consisted of a standardized neurological, neuro-ophthalmological, and neuro-otological examination. This included the head-thrust test, evaluation for spontaneous nystagmus with Frenzel’s googles in the primary position and during gaze deviation, smooth pursuit, saccades, optokinetic nystagmus, visual fixation suppression of the vestibulo-ocular reflex (VOR), head-shaking nystagmus, and determination of the subjective visual vertical (SVV). Deviations above 2.5° were set as pathological (9). Cerebellar function was evaluated by ataxia rating scales, namely, the Scale for the Assessment and Rating of Ataxia (SARA) (10) and the Spinocerebellar Ataxia Functional Index (SCAFI) (11). During the 3-month follow-up, vestibular function was examined neurophysiologically with bithermal caloric testing using the EyeSeeCam System^®^ (pathological side difference according to Jongkee’s formula higher than 25%) and air-conducted cervical and bone-conducted ocular vestibular-evoked myogenic potentials (c/oVEMP) using the Interacoustics Eclipse System^®^ (12). The asymmetry ratio [AR = ((larger-smaller response)/(larger + smaller response)) × 100], should be under 35% (13). Posturographic measurements were carried out in the upright position using the standard protocol with 10 different conditions with increasing difficulty (14). Subjective improvement was measured with an interview. Patients were asked regarding all complaints together whether they had an improvement of their symptoms 3 months after intervention. Subjective impairment with regard to vertigo and dizziness was measured with the Dizziness Handicap Inventory (DHI) (15, 16). The DHI is a self-assessment inventory with a minimum score of 0 and a maximum score of 100 (maximum impact of dizziness on daily life). The clinically relevant change score between pre- and posttreatment in the original work is 18 (16). All patients had routine postoperative MRI after 3 months. In the case of existing syrinx, the size (length and diameter in millimeters) was measured and compared with preoperative MRI scans.

### Ethic Standard Protocol Approvals, Registration, and Patient Consent

This study was carried out in accordance with the recommendations of the Institutional Review Board of the ethics committee of the Ludwig-Maximilian University Munich with written informed consent from all subjects. All subjects gave written informed consent in accordance with the Declaration of Helsinki. The protocol was approved by the Institutional Review Board of the ethics committee of the Ludwig-Maximilian University Munich. Indication for surgical intervention was provided independent of participation in this study.

### Neurosurgical Intervention

Patients underwent intraoperative electrophysiological monitoring for lower cranial nerves, motor-evoked potentials, and somatosensory-evoked potentials in order to avoid an additional effect on the brainstem as a result of exaggerated head inclination. In the prone position, the patient’s head was fixed in a Mayfield clamp. Surgical intervention consisted of a suboccipital medial dissection of the neck muscle exposing the arch of C1 and the occiput around the foramen magnum. First, the foramen magnum was enlarged by osteoclastic craniectomy of approximately 3 cm × 4 cm, the width adapted to the size of the foramen magnum. Thereafter, a laminectomy of the arch of C1 was performed to the same width of the spinal canal. Intraoperative ultrasound allowed the visualization of the tips of the tonsils in order to decompress as far down as necessary. Sometimes a laminotomy of the upper rim of the C2-arch was necessary. C2 laminectomy was never performed in order to avoid cervical instability. Then, the dura was opened in a “Y” fashion and stitched to the periosteum of the occiput. After careful dissection of the arachnoid, the longer and larger cerebellar tonsil as well as the lower brainstem and the spinal cord were identified. The identified tonsil was first shrunk by coagulation of the outer and medial surface without affecting intracranial vessels. The pia of the coagulated tonsil was incised and tonsillar tissue removed by gentle suction and bipolar coagulation. Finally the reduced tonsil laid at the level of the foramen magnum. The mean volume of tonsillar tissue resected ranged from 0.6 to 2.7 cm^3^ (mean 1.4 cm^3^, SD 0.6 cm^3^). Finally, the region of the obex was inspected to provide free outflow of the central canal within the spinal cord whenever safely possible. To further enlarge the craniocervical space, a dural substitute (Lyoplant^®^, B. Braun) was implanted and sutured in a watertight fashion. Finally, the wound was meticulously closed in several layers, e.g., muscles, fascia, subcutis, and cutis to prevent CSF leakage.

### Statistical Analysis

Statistical analysis was performed using SPSS Statistics 20 (IBM, Armonk, NY, USA). As data were not normally distributed, non-parametric paired testing was performed using the Wilcoxon matched pairs test to compare two related samples and the Mann–Whitney *U*-test for between-group comparisons. Differences were considered significant if *p* < 0.05. This study design was exploratory.

## Results

### Patient Characteristics and Symptoms

**Figure 1:**
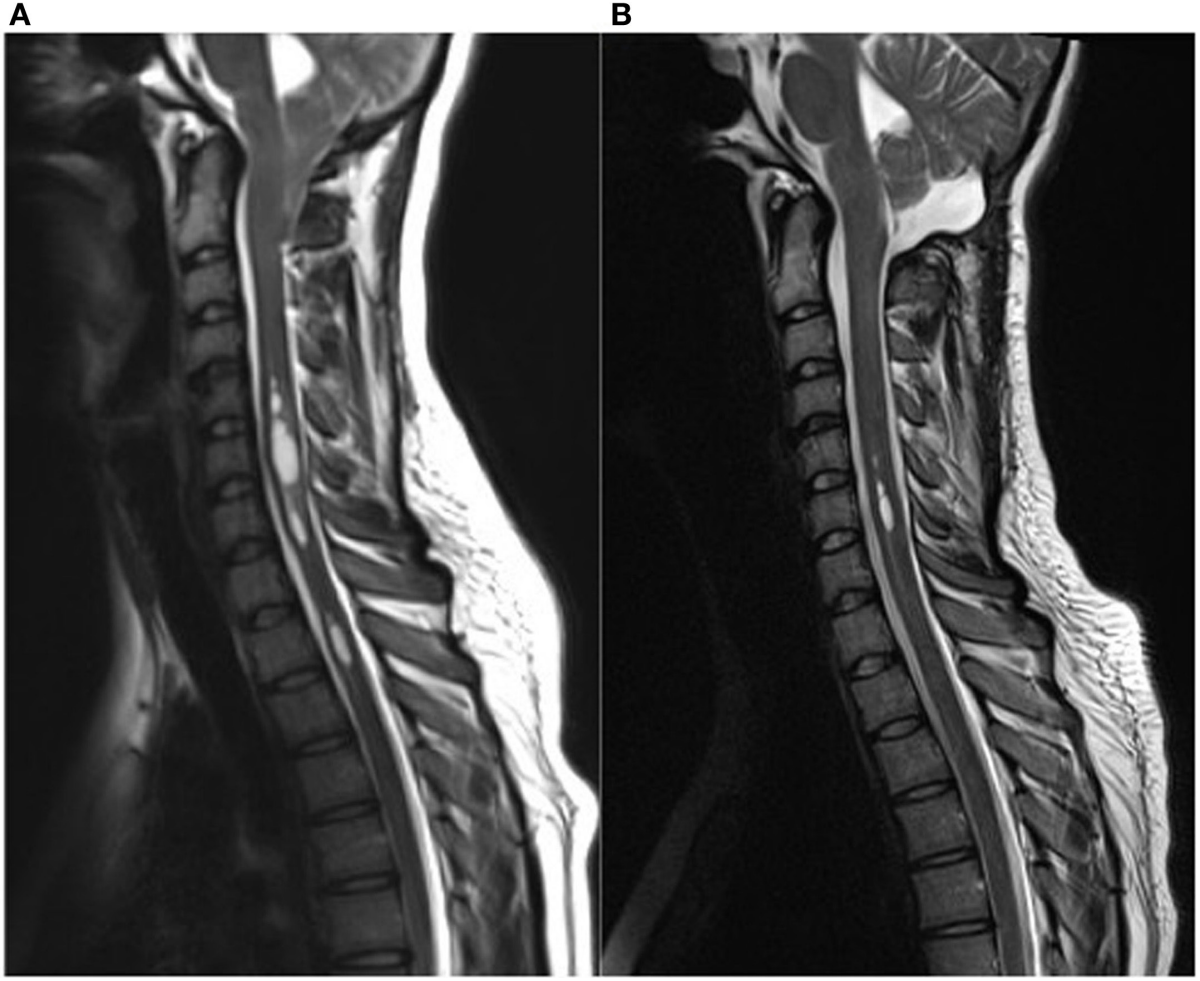
Syringomyelia due to Chiari type I malformation pre **(A)** and 3 months post **(B)** surgical decompression. **(A)** This 19-year-old female complained about occipital headache, hypesthesia of the upper limbs, as well as vertigo attacks. Preoperative sagittal T2-weighted MR images demonstrate tonsil descent down to the lower margin of the arch of C2 and a significant multi-compartmental syringomyelia in the cervical spinal cord. **(B)** Postoperative MR images clearly highlight a marked reduction of preexisting syrinx as well a reduced tonsillar length at the foramen magnum. The patient reported a remarkable improvement of the symptoms after surgical decompression.

Ten patients with a Chiari malformation type 1 confirmed by MRI (6 females, mean age 37 years, range 18–57) were included in this study. The following values are mean values ± standard deviation. Participants were examined preoperatively (27 ± 48 days before surgery; range 1–day–5 months, *n* = 10) and at two time points after surgical intervention [time point 1 (8 ± 4 days after surgery; range 4–16 days, *n* = 6 patients) and time point 2 (4 ± 1 months after surgery; range 3–7 months, *n* = 10 patients)]. For further details on clinical data, see Table 1. In our cohort, the mean duration of symptoms was 4 ± 3 years (range 3 months–11 years). The time between symptom onset and correct diagnosis was 3 ± 3 years (range 3 months–10 years). In MRI, syringomyelia was present in 70% of the patients. Tonsillar descent ranged from 0.7 to 2.2 cm (mean 1.4 cm, SD 0.6 cm). The length of the syringomyelia varied between one and more than 10 spinal segments, the size between 1.5 and 0.3 mm in maximal diameter. Figure 1 shows patient number four with a significant syringomyelia due to Chiari type I malformation pre- and postoperatively.

**Table 1:** Clinical presentation and course after surgical intervention.

P	Age	Clinical presentation before the intervention	Duration of symptoms (months)	Syrinx	Volume of tonsil (ccm^3^)	Volume of tonsillar resection (ccm^3^)	Side of res. tonsil	Time (months)	Clinical presentation after the intervention
1	40–45	Headache, vertigo attacks, **dysphagia, hoarseness of the voice**, dysesthesia in the hands	4	y	1.8	1	r	3	Free of complaints
2	18–20	**Headache**, attacks of dizziness, dysphagia	24	y	1.2	0.6	r	4	Free of complaints
3	50–55	Dysesthesia in the hands, **headache, and back pain**	84	y	1.9	1	l	3	Hypaesthesia in the fingers
4	50–55	**Pain in the upper limbs, paresis, and atrophy of hand muscles**	36	y	1.5	0.8	l	3	Improvement of paresis and atrophy of hand muscles
5	55–60	**Vertigo** attacks	48	y	2.2	1.6	l	5	Pain in the right hand
6	35–40	Vertigo attacks, **headache**, dysphagia, hypacusis	3	n	1.7	0.9	l	3	Improvement of headache, dizziness during fixation
7	20–25	**Vertigo attacks, neck pain**, hypesthesia, and decreased fine motor skills of the hands	36	y	3.1	1.8	l	7	Vertigo during head movements
8	35–40	**Double vision**, dizziness attacks	132	n	4.6	2.7	r	3	Unchanged
9	20–25	**Headache**	15	n	2.5	1.7	l	5	4/5 paresis and hyperesthesia of the right upper limb
10	25–30	**Paresis and atrophy of hand muscles** and decreased fine motor skills, dysesthesia in the hands	36	y	2.5	1.7	l	3	Improvement of paresis and atrophy of hand muscles

*The leading symptoms that led to consultation of a doctor are printed in bold type*.

*P, patient number; duration of symptoms, duration of the symptoms before intervention in months; y, yes; n, no; volume of tonsil, volume of the tonsil before surgery in ccm^3^; volume of tonsillar resection, volume of the resected tonsil in cm^3^; Side of res. Tonsil, side of resected tonsil; l, left; r, right; time, time after the postoperative intervention for the examination (months)*.

The main symptoms were recurrent attacks of vertigo or dizziness (60%), headache, and neck pain, which worsened during coughing and specific head movements (50%) and dys-/hypesthesia in upper limbs (50%).

In terms of clinical–neurological characteristics, the main findings were ataxia in the upper limbs (finger-chase dysmetria, intention tremor, dysdiadochokinesis) (6/10 patients) and dys- and hypaesthesia (5/10 patients). For further details of the clinical examination, see Table 2 (A).

**Table 2:** Ocular motor (A) and neurological findings (B) before and after surgical intervention.

	A			B		
P	Pathological ocular motor findings			Pathological neurological findings		
	Before	After 1 week	After 3 months	Before	After 1 week	After 3 months
1	GEN r > l, PN l, HIT r	SP v and h	SP v and h	Finger-chase dysmetria b, Heel-shin slide dysmetria, dysesthesia in both hands	Finger-chase dysmetria l	Finger-chase dysmetria l
2	Strabismus sursoadductorius l	md	Unchanged	None	md	None
3	n	md	n	Finger-chase dysmetria l, Hypaesthesia l hand and arm	md	None
4	PN l	n	DBN	Atrophy and paresis of the interosseal muscles, finger-nose dysmetria l	Gait ataxia, atrophia and paresis unchanged, finger-nose dysmetria r > l	Improvement of paresis and atrophy of hand muscles, Finger-chase dysmetria l
5	n	SVV r	n	Unsteady gait, hypesthesia right arm and leg	Unsteady stance, finger-chase dysmetria and diadochokinesis r	Finger-chase dysmetria
6	OTR r (head tilt, SVV), VFS v	n	n	Intention tremor l	Finger-chase dysmetria l	Hearing impairment l, finger-chase dysmetria l
7	n	n	SP v	Dysdiadochokinesis r, hypesthesia in both hands, pallhypesthesia	none	Lateral canal BPPV r
8	GEN h, RN, SP v and h, hypermetric saccades v and h, VFS h > v, HIT l and r, PN l, DBN	GEN h, RN, SP v and h, hypermetric saccades v and h, VFS h > v, PN l	GEN h, RN, SP h and v, hypermetric saccades h, VFS h > v, SVV l	Isolated ocular motor findings	md	Unchanged
9	n	md	GEN h, RN, SP v	None	md	4/5 paresis and hyperesthesia of the right upper and lower limb with reduced reflexes, dysdiadochokinesis r
10	n	SVV r	SP h	Paresis of triceps and interosseal muscles, finger-nose dysmetria l >r, hypesthesia in the fingers	md	Improvement of the paresis and hypesthesia

*P, patient number; r, right; l, left; b, both; h, horizontal; v, vertical; n, none; md, missing data; GEN, Gaze-evoked nystagmus; PN, head-shaking nystagmus; HIT, head impulse test; SP, smooth pursuit; DBN, downbeat nystagmus; SVV, deviation of the subjective visual vertical > 2.5°; OTR, ocular tilt reaction; VFS, visual fixation suppression of the vestibulo-ocular reflex; RN, rebound nystagmus*.

In the neuro-ophthalmological examination, five patients had abnormal ocular motor findings including head-shaking nystagmus (*n* = 3), gaze-evoked-nystagmus, disturbed fixation suppression of the VOR, pathological head impulse test (each *n* = 2), deviation of SVV, head tilt, hypermetric saccades, rebound nystagmus, saccadic smooth pursuit, and downbeat nystagmus (DBN) (each *n* = 1). For further information, see Table 2 (B).

Patients had mild cerebellar deficits in the examined scores. The mean total SARA score was 0.6 ± 0.9 (the score ranges from 0 = no ataxia to 40 = severe ataxia). Mean 8MW was 5.1 ± 0.5 s. 9-Hole peg test (9HPT) of the dominant hand was 20.1 ± 2.2 s and of the non-dominant hand 21.3 ± 2.6 s. The mean PATA rate was 27.9 ± 3.9. For further information see Table 3.

**Table 3:** Cerebellar function evaluated by the Scale for the Assessment and Rating of Ataxia (SARA) and the Spinocerebellar Ataxia Functional Index (SCAFI) before and after surgical intervention.

P	SARA			SCAFI 8MW (s)			SCAFI 9HPT D (s)			SCAFI 9HPT ND (s)			SCAFI PATA		
	Before	After 1 week	After 3 months	Before	After 1 week	After 3 months	Before	After 1 week	After 3 months	Before	After 1 week	After 3 months	Before	After 1 week	After 3 months
1	1 (b^a^)	0.5 (l^a^)	0.5 (l^a^)	5.5	5.3	5.0	23.0	21.5	23.3	25.5	26	24.0	31.0	26.0	26.5
2	0	md	0	5.3	md	4.6	24.1	md	19.9	23.0	md	21.0	27.0	md	25.0
3	0.5 (l^a^)	md	0	5.0	md	4.0	19.0	md	18.3	20.2	md	21.0	24.0	md	37.0
4	0.5 (l^a^)	3 (^a^^,^^b^^,^^c^)	0.5 (l^a^)	4.5	4.8	3.6	19.0	21.7	18.1	24.3	23.2	23.5	20.5	23.5	25.0
5	0	2	0.5 (r^a^)	5.9	5.5	5.7	20.4	17.6	19.5	22.6	21.0	23.8	33.0	32.0	30.0
6	0.5 (l^d^)	0.5 (l^a^)	0.5 (l^a^)	4.7	md	4.1	18.3	md	16.4	18.1	md	17.5	26.0	md	30.5
7	0.5 (r^e^)	0	0	4.9	6.6	4.8	18.1	19.2	16.1	19.8	18.8	16.5	26.5	30.0	29.0
8	0	md	0	5.8	md	4.0	17.5	md	17.4	17.3	md	16.9	31.5	md	36.5
9	0	md	0.5 (r^e^)	4.5	md	4.7	20.6	md	19.7	21.4	md	20.1	31.5	md	32.0
10	3 (^a^^,^^e^)	md	0	5.3	md	5.3	21.3	md	21.9	21.0	md	20.6	28.0	md	28.0
MW	0.6	1.2	0.3	5.1	5.5	4.6	20.1	20.0	19.0	21.3	22.2	20.5	27.9	27.9	30.0
SD	0.9	1.3	0.3	0.5	0.8	0.6	2.2	2.0	2.3	2.6	3.1	2.8	3.9	3.8	4.3

*SARA consists of a clinical evaluation of eight items: gait, stance, sitting, speech disturbances, finger-chase, nose-finger-test, fast alternating hand movements, and heel-shin slide. SCAFI is composed of a timed 8 m walk at maximum speed (8MW), the 9-hole peg test (9HPT) for fine motor skills, and the PATA rate, a measure of speech performance*.

*P, patient number; l, left; r, right; b, both; md, missing data; 9HPT D, 9-hole peg test dominant hand; 9HPT ND, 9-hole peg test non-dominant hand*.

*^a^Finger-chase dysmetria*.

*^b^Gait ataxia*.

*^c^Stand ataxia*.

*^d^Tremor in the nose-finger test*.

*^e^Irregularities in fast alternating hand movements*.

Nine out of 10 patients underwent caloric testing before surgical intervention, eight o/cVEMP. Six patients had abnormal vestibular function in these tests: three patients (no. 2, 3, and 7) had a pathological side difference in caloric testing, suggesting a deficit in horizontal canal function; three other patients had pathological o/cVEMP testing, indicating a deficit in otolith function (no. 1, 9, and 10).

The total mean DHI was 17 (range 0–70), suggesting a mild handicap. For further information, see Table 4.

**Table 4:** Vestibular testing of horizontal canal (caloric testing), saccular [cervical ocular evoked myogenic potentials (cVEMP)], and utricular [ocular evoked myogenic potentials (oVEMP)] function as well as subjective impairment, measured by the Dizziness Handicap Inventory (DHI), before and 3 months after surgical intervention.

P	Caloric						cVEMP						oVEMP						DHI							
	Before			After 3 months			Before			After 3 months			Before			After 3 months			Before				After 3 months			
	Max. SPV (°/s)		AR (%)	Max. SPV (°/s)		AR (%)	Max. Amp. (µV)		AR (%)	Max. Amp. (µV)		AR (%)	Max. Amp. (µV)		AR (%)	Max. Amp. (µV)		AR (%)								
													
	r	l		r	l		r	l		r	l		r	l		r	l		p	e	f	t	p	e	f	t
1	14	14	2	13	14	2	nr	nr	nr	95	163	26	28	29	2	144	116	11	6	22	26	54	2	0	2	4
2	3	11	54	7	21	50	442	268	25	33	31	3	39	29	15	md	md	md	0	0	0	0	0	0	0	0
3	15	8	28	14	11	13	69	83	9	33	39	8	27	26	2	10	12	9	0	0	0	0	0	0	0	0
4	22	19	10	20	29	19	84	57	19	89	150	26	31	18	27	53	62	8	0	0	0	0	0	0	0	0
5	23	28	10	md	md	md	38	50	14	md	md	md	45	32	17	md	md	md	0	0	0	0	0	0	0	0
6	md	md	md	md	md	md	md	md	md	30	27	5	md	md	md	50	54	4	24	24	22	70	0	0	0	0
7	15	8	32	19	5	57	61	118	32	28	54	32	9	9	0	6	3	33	6	12	10	28	6	8	8	22
8	37	38	1	41	41	1	md	md	md	53	md	md	md	md	md	md	md	md	md	md	md	md	md	md	md	md
9	13	11	7	4	8	34	144	67	36	35	32	4	43	27	23	19	19	0	4	0	0	4	6	14	10	30
10	36	28	13	37	28	15	294	215	16	96	121	12	17	7	42	md	md	md	0	0	0	0	0	0	0	0

*Pathological values as described in the Section “Materials and Methods” have a gray background*.

*P, patient number; Max. SPV, maximal slow phase velocity; AR, asymmetry ratio; r, right; l, left; p, physical subscale of the DHI; e, emotional subscale of the DHI; f, functional subscale of the DHI; t, total DHI; md, missing data; nr, not reproducible*.

Patients had a tendency to poorer performance in posturography compared to healthy controls published in other studies in our center (17). Standing on firm ground, nine out of 10 patients showed a sway pattern similar to phobic postural vertigo in artificial network (14). For further information, see Table 5.

**Table 5:** Extract of posturography data with total and directional sway, root mean square (RMS), as well as fast Fourier transformation (FFT) before and after surgical intervention in four selected examinations.

		Eyes open, head straight		Eyes closed, head straight		Eyes open, foam ground		Eyes open, tandem stand	
		Before	After 3 months	Before	After 3 months	Before	After 3 months	Before	After 3 months
FFT 3.5–8 Hz *X*	mm/Hz	719.1	580.2	891.5	631.2	1,196.0	1,101.4	2,169.6	2,107.5
*Y*	mm/Hz	961.3	787.6	1,318.7	1,025.0	1,433.7	1,326.3	3,147.2	3,154.8
*Z*	N/Hz	45.1	37.0	47.6	44.2	51.2	51.7	89.3	93.1
RMS (total)	mm	7.5	8.9	7.7	7.6	12.6	12.7	17.5	17.7
*X*	mm	3.9	4.3	3.8	3.6	8.2	8.8	11.9	11.7
*Y*	mm	6.2	7.4	6.6	6.7	9.4	8.9	12.6	13.2
*Z*	Mm	0.01	0.01	0.01	0.01	0.02	0.02	0.04	0.04
Sway (total)	m/min	1.1	1.1	1.5	1.2	1.7	1.5	3.3	3.1
*X*	m/min	0.7	0.6	0.8	0.6	1.0	0.9	1.8	1.6
*Y*	m/min	0.8	0.8	1.1	0.9	1.2	1.1	2.4	2.3
*Z*	m/min	0.02	0.02	0.02	0.02	0.02	0.02	0.04	0.04

*There was no significant difference between the different time points before and after the intervention*.

### Symptoms, Clinical Signs, and Findings 1 Week after Surgery

Short-term follow-up examination was performed in five patients. New documented deficits included finger-chase dysmetria (no. 4, 5) and gait ataxia (no. 4). This could be confirmed in ataxia scores (SARA 0.6 ± 0.9 to 1.2 ± 1.3, SCAFI 8MW 5.1 ± 0.5 to 5.5 ± 0.8 s).

Neuro-ophthalmological examination revealed a deviation of the SVV to the right after resection of the left tonsil (patient no. five 5.2° and patient no. ten 4.5°) and new smooth pursuit deficits (no. 1). For further information, see Table 2.

### Symptoms, Clinical Signs, and Findings 3 Months after Surgery

Sufficient suboccipital decompression led to improvement of symptoms (measured with an interview regarding all symptoms before intervention) in 9/10 of patients in follow-up without influence of the side of the resected tonsil. Two patients were free of complaints after the neurosurgical intervention (no. 1, 2). Symptoms improved in seven patients (no. 3–8, no. 10). One patient had major complications due to CSF leakage and subsequent meningitis. He needed surgical revision for dural leak, and symptoms improved slowly over several weeks (no. 9). In follow-up, he had a residual mild right-sided hemiparesis. There were no other complications in any of the other patients. The size and length of the preexisting syringomyelia decreased in all patients. For further information, see Tables 1 and 2 (B).

Especially hyp-/dysesthesia (5/5 patients), ataxia of the upper limbs (4/5 patients), and paresis of triceps and interosseal muscles (2/2 patients) improved in the follow-up examination after surgical intervention. Fine motor skills and gait improved significantly [SCAFI 8MW from 5.1 ± 0.5 to 4.6 ± 0.6 s; *p* = 0.018; 9HPT from 20.7 ± 2.2 to 19.8 ± 2.3; *p* = 0.028 (dominant hand: 20.1 ± 2.2 to 19.0 ± 2.3, *p* = 0.022; non-dominant hand: 21.3 ± 2.6 to 20.5 ± 2.8, *p* = 0.093)]. Speech measured with the PATA test remained unchanged (27.9 ± 3.8 to 30.0 ± 4.3 words in 10 s, *p* = 0.21). The same applies to the SARA score (0.6 ± 0.9 to 0.3 ± 0.3, *p* = 0.317). For further information, see Table 3.

Neuro-ophthalmologically, head-shaking nystagmus, VOR, ocular tilt reaction, and DBN improved in all affected patients. A partial improvement could be observed in gaze-evoked nystagmus and fixation suppression of the VOR.

New ocular motor disturbances after surgery were smooth pursuit deficits (no. 1, 7, 9, 10), DBN (no. 4), and gaze-evoked nystagmus and rebound nystagmus (no. 9). All of these patients were asymptomatic. Patient number 9 with major postoperative complications had further new vestibulocerebellar dysfunctions, including gaze-evoked nystagmus, rebound nystagmus, vertical smooth pursuit deficit, and vestibular deficit in the caloric testing and reported more impairment in the DHI. For further information, see Table 2 (A) and Table 4.

The two patients with pathological cVEMP improved, as did one out of three patients with abnormal caloric testing. The mean DHI improved from 17 to 6 after surgical intervention (*p* = 0.273), especially based on two patients (no. 2, 6), who had a significant improvement of impairment due to vertigo and dizziness. For further results, see Table 4.

In posturographic measurements after surgery, total sway (and RMS values) did not change significantly. There was no typical cerebellar 3 Hz-sway. For further information, see Table 5.

## Discussion

The major findings of this prospective study are as follows.

First, preoperative neuro-opthalmological examination revealed abnormal findings in 50% of patients, including head-shaking nystagmus, gaze-evoked-nystagmus, disturbed fixation suppression of the VOR, deviation of the SVV, head tilt, hypermetric saccades, rebound nystagmus, saccadic smooth pursuit, and DBN. 70% of patients had abnormal vestibular function: two patients had a pathological head impulse test indicating a high-frequency deficit of the VOR, three patients had a pathological caloric test testing the low-frequency function of the VOR and three had abnormal otolith function tested with vestibular-evoked myogenic potentials.

Patients had only mild to moderate cerebellar signs (especially finger-chase dysmetria). The clinical symptoms of our Chiari patients were similar to those in other studies (1, 2, 18) except for higher vestibular symptoms supporting former results that detection is higher in departments specialized in neuro-otology (6). In a study with 77 Chiari-patients in a specialized department over several years, 55% of the patients showed preoperative abnormalities in the examinations of the ocular motor system, including horizontal spontaneous nystagmus (27%), saccadic smooth pursuit (29%), positional induced nystagmus (21%), saccadic dysmetria (10%), and DBN (7%) (18). The large number of patients with spontaneous horizontal nystagmus could not be reproduced by our data, even though three patients (39%) had a horizontal head-shaking nystagmus, suggesting that the velocity storage was affected.

Second, surgical intervention including tonsillectomy led to subjective and objective improvement of the symptoms in 9/10 of patients. Fine motor skills and gait measured with SCAFI improved significantly. Impaired vestibular (horizontal canal, saccular, and utricular) function improved in five of seven patients with impaired function before surgery, especially in terms of high-frequency VOR function and saccular otolith function. Ocular motor disturbances improved in all affected patients in terms of head-shaking nystagmus, VOR, ocular tilt reaction, and DBN. In some patients, further improvement could be observed in terms of gaze-evoked nystagmus and fixation suppression of the VOR.

The subjective improvement in our series exceeds that of case series and reviews which generally show an improvement of around 75% (3). However, tonsillar resection was described in only 27% of patients with suboccipital decompression according to a review on a surgical series between 1965 and 2013 (3). In another series of 177 patients, who underwent posterior fossa surgery, the 137 patients who underwent tonsillar resection with subsequent larger subarachnoid cisterns showed a more pronounced reduction in the size of syringomyelia and subsequently a greater improvement in their symptoms (1). In another observational study on 77 patients, tonsillar resection had good results in 100% compared to 64.8% in those with only foramen magnum decompression (4). In a large series of 371 patients using posterior fossa decompression without tonsillar resection, an improvement of preoperative symptoms was described in only 73.6% of patients (19).

Third, neuro-ophthalmological examination after suboccipital decompression, including tonsillar reduction, revealed new, but mostly asymptomatic, ocular motor deficits in five patients, including smooth pursuit deficits, horizontally gaze-evoked nystagmus, rebound nystagmus, and DBN. Contralateral (to the side of tonsillar reduction) deviation of the SVV was temporary in 20% of patients and was compensated in follow-up. Previous retrospective studies about vestibulo-ocular manifestations in Chiari malformation or syringomyelia showed a remarkable resolution of ocular motor disorders of 75–100% after decompression without tonsillectomy (5–7). The surgical procedure in these series consisted of decompression of the foramen magnum and C1 laminectomy including duroplasty without tonsillar resection. On the other hand, the ocular motor examination, which was not performed by specialized neuro-opthalmologists, was not as detailed and standardized as in our series.

So far there have been no publications about ocular motor, cerebellar, and vestibular side effects of suboccipital decompression with unilateral reduction of the cerebellar tonsil, which is considered in neuroanatomical studies to be an important part of the vestibulocerebellum. In animal experiments, surgical lesions of the flocculus and paraflocculus led to impaired smooth pursuit, horizontally gaze-evoked nystagmus, rebound nystagmus, and DBN (20).

In our study, we described new ocular motor deficits after suboccipital decompression with reduction of one cerebellar tonsil, including smooth pursuit deficits, horizontal gaze-evoked nystagmus, rebound nystagmus, and DBN as well as contralateral deviation of the SVV. As described, these findings are consistent with animal studies. A case report describing a patient with acute isolated unilateral infarction of the cerebellar tonsil showed impaired smooth pursuit, an ipsilateral nystagmus, gaze-evoked nystagmus, and a mild contralateral tilt of the SVV (8). In summary, our results demonstrate that the human tonsil mainly seems to contribute to smooth pursuit and the gaze holding network (8). This is supported by anatomical connectivity studies, as the paraflocculus mainly receives input from the contralateral pontine nuclei (21), the contralateral inferior olivary nucleus, and the paramedian tract, suggesting an important function in the adaptive ocular motor control and the neural integrator network (22).

Why are the deficits after a unilateral tonsillectomy so moderate compared to clinical findings even after small tonsillar infarctions? A likely explanation could be that the function has already been impaired due to compression and this deficit has already been compensated centrally. In addition, the size of the perioperative tonsillar lesion was smaller.

We are aware of the limitations of our study. First, the design is prospective but not controlled. Intraoperative neurosurgical decisions, i.e., on the side of the reduced tonsil, were not randomized. The number of patients is rather small (*n* = 10) compared to retrospective studies (6, 18). Our results were based on examination by specialized orthoptists. Most of the new ocular motor dysfunctions were mild and only detectable in detailed ocular motor examination and therefore might be missed in other studies. Another limitation is the time range in which the postoperative follow-up examinations took place. As the cerebellum is known to have a remarkable compensatory capacity, this might have influenced our results. During the first postoperative examination, two patients were taking tilidine as a painkiller, which is known to affect smooth pursuit (23). Therefore, medical interactions cannot be ruled out at this time. This study was exploratory with no correction for multiple testing, which could have influenced the significance of the SCAFI test.

Suboccipital decompression including reduction of one cerebellar tonsil can lead to similar ocular motor deficits as in ischemic infarctions; however, nearly all of our patients were asymptomatic. With a subjective and clinical improvement in 9/10 of our patients, we can promote the resection of one cerebellar tonsil if the size of the tonsil or the degree of downward displacement makes it necessary. Neuro-anatomically, our results confirm the role of the tonsil in smooth pursuit and the gaze holding network (8, 24). However, our study includes a complex neurosurgical procedure with decompression of the suboccipital region including tonsillar reduction, which excludes precise anatomical localization. Vestibular deficits indicate a tone imbalance for the semicircular canal and otolith system, which is typically localized in the flocculus or brainstem (25). In summary, this is the first prospective clinical study about the effect of unilateral cerebellar tonsil resection on ocular motor, vestibular and cerebellar function. Surgical decompression, including unilateral cerebellar tonsil reduction/resection, leads to a good clinical outcome in terms of patients’ complaints, fine motor skills, and gait without any effect of the side of the resected tonsil.

Nevertheless, a detailed postoperative examination can show ocular motor disorders similar to those resulting from ischemic infarctions (smooth pursuit deficits, horizontally gaze-evoked nystagmus, rebound nystagmus, and DBN as well as deviation of the SVV), confirming the anatomical role of the tonsil in smooth pursuit and the gaze holding network; however, symptoms are mild and asymptomatic in our Chiari patient cohort.

## Ethics Statement

This study was carried out in accordance with the recommendations of the Institutional Review Board of the ethics committee of the Ludwig-Maximilian University Munich with written informed consent from all subjects. All subjects gave written informed consent in accordance with the Declaration of Helsinki. The protocol was approved by the Institutional Review Board of the ethics committee of the Ludwig-Maximilian University Munich. Indication for surgical intervention was provided independent of participation in this study.

## Author Contributions

NG: research project (conception, organization, execution, interpretation of the data), statistical analysis (design, execution), manuscript preparation (writing of the first draft, review). KF: research project (conception, organization, execution), manuscript preparation (review and critique). FI and MK: research project (execution), manuscript preparation (review). SK: research project (interpretation of the data), manuscript preparation (review). JT: research project (idea for study, conception, interpretation of the data), manuscript preparation (review and critique). MS: research project (idea for study, conception, interpretation of the data), manuscript preparation (review and critique) including medical writing for content. AP: research project (conception, organization, execution, interpretation of the data), manuscript preparation (review and critique) including medical writing for content.

## Conflict of Interest Statement

The authors declare that the research was conducted in the absence of any commercial or financial relationships that could be construed as a potential conflict of interest.
