# Supplementary material for: Decompression in Chiari Malformation: Clinical, Ocular Motor, Cerebellar, and Vestibular Outcome
Source: Front Neurol. 2017 Jun 22;8:292. doi: 10.3389/fneur.2017.00292 (PMC5479925; doi:10.3389/fneur.2017.00292)
Supplement: Supplementary file 1 [file Data_Sheet_1.DOCX]

Supplementary Material

**Supplementary Data e-1**

**Historical overview of surgical correction of Chiari malformation**

**Nicolina Goldschagg MD^1,2^, Katharina Feil MD^1,2^, Franziska Ihl^1^, Siegbert Krafczyk^2^, Mathias Kunz MD^3^, Jörg Christian Tonn MD^3^, Michael Strupp MD, FANA, FEAN^1,2,^ Aurelia Peraud MD^3^**

^1^German Center for Vertigo and Balance Disorders, University Hospital Munich, Munich, Germany

^2^Department of Neurology, University Hospital Munich, Munich, Germany

^3^Department of Neurosurgery, University Hospital Munich, Munich, Germany

***Corresponding author:**

Nicolina Goldschagg, MD

Email: nicolina.goldschagg@med.uni-muenchen.de

**1 Supplementary Data e-1:**

**Historical overview of surgical correction of Chiari malformation**

Chiari malformations were first described by Hans Chiari (1851-1916) a pathologist in Prague and Strasbourg. His initial work was published in the Deutsche Medizinische Wochenschrift in 1891 with the title “Concerning alterations in the cerebellum resulting from cerebral hydrocephalus”. The Chiari Type I malformation was characterized by “elongation of the tonsils and medial divisions of the inferior lobules of the cerebellum into cone shaped projections which accompany the medulla oblongata into the spinal canal”. He further outlined “the elongated portions of the cerebellum can show either normal structure, fibrosis or softening ... and ... extend nearly to the top of the atlas, however in many cases to the undersurface of the axis”. Five years later he described 14 cases with type I changes and found that the grade of hydrocephalus was not related to the severity of craniospinal changes and concluded that an additional mechanism may play a role in this condition (“insufficient bone growth with insufficient enlargement of skull parts resulting in increased ICP”).

He further described other malformations, with Type II being characterized by a displacement of parts of the inferior vermis, pons, and medulla oblongata as well as elongation of the fourth ventricle into the spinal canal. A few other authors, such as Cleland and Arnold, had already published on this malformation. They reported on pediatric cases with spina bifida and hydrocephalus similar to Chiari Type II.

The first report on surgical correction of this deformity was done by Van Houwenige Graftidijk in his medical thesis “On hydrocephalus” in 1932. His intention was to relieve CSF flow obstruction at the level of the deformity by resection of the tongue of the herniated tissue and the bone over the posterior surface including an incision of the dura. Others followed with descriptions of surgical results. Penfield and Coburn in 1938 reported on a 29-year-old woman and thoracic spina bifida in infancy. The patient died 2 months later and at autopsy a Chiari Type II malformation with hydrocephalus was found. They suggested leaving the cerebellar tonsils intact and removing the posterior margin of the foramen magnum and C1 arch. Also in 1938 McConell and Parker published their successful results on five Chiari Type I patients, as did Bucy and Lichtenstein in 1945 and Chorobski and Stepien in 1948. According to the published results of Gardner and coworkers in 1950 the mortality was as high as 12% and this did not change over the following years even up to the 1970s. However the studies included only a relatively small number of patients with a short follow-up. The purpose of Klekamp’s review in 2011 was to evaluate the risks and benefits of a larger group of patients (n=371) who underwent a foramen magnum decompression in three different neurosurgical clinics from 1985 to 1991. The surgical procedure included a small bony decompression of the foramen magnum, a medial laminectomy of C1, arachnoid dissection with coagulation of the tonsillar tips to inspect the foramen of Magendie in order to open of the fourth ventricle and a duraplasty with alloplastic material. He concluded that this technique yields excellent results when performed as the first procedure. However, severe arachnoid scaring and fibrosis seem to be responsible for the lower success rate in secondary decompressions.
